# Auto-injector needle length may be inadequate to deliver epinephrine intramuscularly in women with confirmed food allergy

**DOI:** 10.1186/1710-1492-10-39

**Published:** 2014-07-22

**Authors:** Gina Tsai, Laura Kim, Immaculate FP Nevis, Arunmozhi Dominic, Ryan Potts, Jack Chiu, Harold L Kim

**Affiliations:** 1 Schulich School of Medicine & Dentistry, Western University, London, Ontario, Canada; 2 Department of Anatomy and Cell Biology, McGill University, Montreal, Quebec, Canada; 3 Michael D. DeGroote School of Medicine, McMaster University, Hamilton, Ontario, Canada; 4 Department of Biology, University of Waterloo, Waterloo, Ontario, Canada; 5 525 Belmont Avenue West, Suite 205, Kitchener, Ontario N2M 5E2, Canada

**Keywords:** Food allergy, Anaphylaxis, Skin-to-muscle depth, Epinephrine, Auto-injector, Needle length

## Abstract

**Background:**

Epinephrine auto-injectors are the standard first aid treatment for anaphylaxis. Intramuscular delivery into the anterolateral aspect of the thigh is recommended for optimal onset of action of epinephrine. The most frequently prescribed auto-injector in North America and Canada is the EpiPen^®^, which has a needle length of 15.2 mm. Currently, it is unknown whether this needle length is adequate for intramuscular delivery of epinephrine in adult patients at risk of anaphylaxis.

**Methods:**

One hundred consecutive adult patients with confirmed food allergy requiring an epinephrine auto-injector were recruited. Skin to muscle depth (STMD) at the right mid-anterolateral thigh was measured using ultrasound under minimal (min) and maximum (max) pressure. The EpiPen^®^ needle length was considered adequate if STMD_max_ was ≤15.2 mm. Baseline characteristics including age, gender, ethnicity, and body mass index (BMI) were compared in patients with STMD_max_ ≤15.2 mm vs. >15.2 mm.

**Results:**

The EpiPen^®^ needle length of 15.2 mm was inadequate for intramuscular delivery in 19 of the 100 enrolled patients (19%), all of whom were female; 28% of women had a STMD_max_ >15.2 mm. The mean STMD_max_ in the ≤15.2-mm and >15.2-mm groups were 9 ± 4 mm and 20 ± 4 mm, respectively (p = 0.0001). Linear regression analysis found BMI to be significantly associated with STMD_max_ after adjusting for age (p < 0.001).

**Conclusions:**

The needle length of the epinephrine auto-injectors may not be adequate for intramuscular delivery of epinephrine in a large proportion of women with food allergy; this may impact morbidity and mortality from anaphylaxis in this patient population.

## Introduction

Anaphylaxis is a serious allergic reaction that is rapid in onset and may cause death [1]. Although there are several causes of fatal anaphylaxis, food allergy is one of the most common [2]. Epinephrine is recommended as the initial treatment of choice for anaphylaxis [1,3]. A delay in epinephrine administration may contribute to an increased risk of death [2]. Previous studies have demonstrated that the site and route of administration of epinephrine significantly affect both its peak concentration and time to onset of action. In children, intramuscular administration of epinephrine resulted in a higher peak concentration and shorter time at which peak concentration was achieved than subcutaneous administration [4]. A subsequent study in adult males showed that peak plasma epinephrine concentrations were significantly higher after intramuscular injection into the thigh than following intramuscular or subcutaneous administration into the upper arm [5]. Therefore, the World Allergy Organization recommends that, for the treatment of anaphylaxis, epinephrine (0.01 mg/kg of a 1:1000 [1 mg/mL] solution to a maximum of 0.5 mg in adults and 0.3 mg in children) be administered intramuscularly in the mid-anterolateral thigh [3].

Outside of the hospital setting, epinephrine auto-injectors have become the mainstay of therapy for patient self-treatment of anaphylaxis. In Canada, the most commonly prescribed epinephrine auto-injector for adult patients is the EpiPen^®^[6], which has a needle length of 15.2 mm [7]. Given the increasing prevalence of obesity in North America, two studies have examined whether the design of available epinephrine auto-injectors is adequate for delivering epinephrine intramuscularly to adult patients [8,9]. Song et al. retrospectively analyzed the skin-to-muscle distance in the anterolateral aspect of the thigh in 50 men and 50 women who had undergone computed tomography (CT) of the thighs for other medical reasons [9]. The investigators found that, in a large proportion of women, the EpiPen^®^ may not deliver epinephrine to the intramuscular tissue. Bhalla et al. prospectively assessed depth-to-muscle measurements of the vastus lateralis in low-acuity general emergency department patients and found that the current epinephrine auto-injector needle length is inadequate for intramuscular administration, particularly in women [8]. The current study was performed to examine whether the needle length of the EpiPen^®^ is appropriate for injecting epinephrine into the intramuscular space in men and women at risk of anaphylaxis from food allergy. To our knowledge, this is the first study of this nature to be conducted in adult patients at risk of anaphylaxis.

## Methods

**Figure 1:**
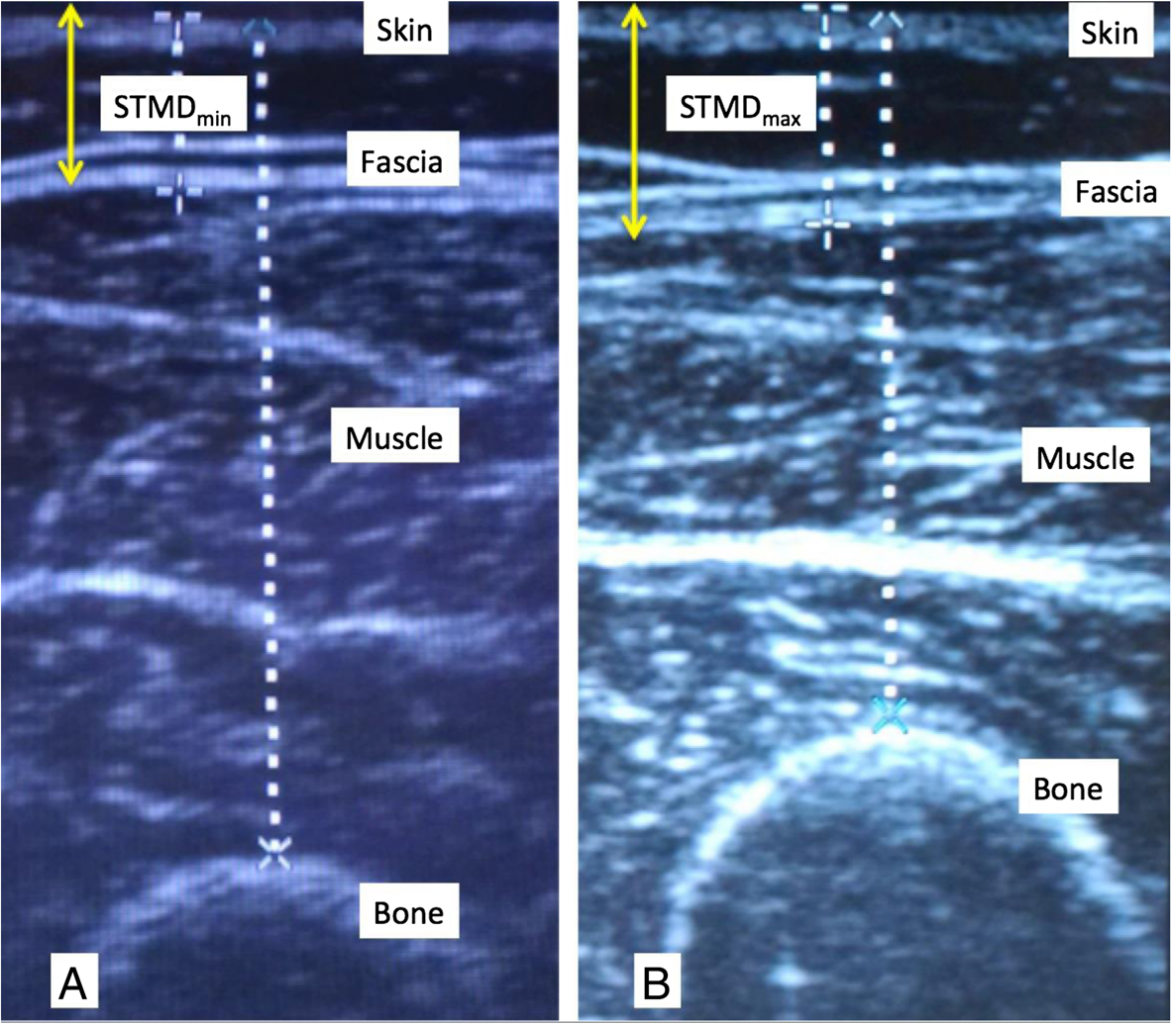
Ultrasound images in a patient with A. minimal pressure and B. maximal pressure.

One hundred consecutive adult patients with confirmed food allergy who required epinephrine auto-injector prescriptions in an allergist’s office were included in this study. The subjects were both new and follow-up patients who were assessed from July 2012 to November 2013. All subjects agreed to participate in the study. Demographic information including patient race, sex, age, weight and height was collected and ultrasound images of the anterolateral aspect of the mid-right thigh were taken in the standing position using the Sonosite Titan^®^ ultrasound machine. Measurements were obtained for the same anatomical location in all patients. Skin-to-muscle depth (STMD) at the right mid-anterolateral thigh was measured by a single operator (a physician) under minimal (min) and maximum (max) pressure. Minimal pressure was defined as “just enough” pressure to obtain an adequate image, and maximum pressure was defined as pressure mimicking that required for proper auto-injection, which is estimated to be approximately 2 to 8 lbs [10]. The physician visualized the soft tissue while performing the compression and froze the screen for measurement when the soft tissue was maximally compressed. Measurements in both the minimal and maximum compression views in frozen screen mode were recorded (Figure 1). Each image screen shot also included the femur for reference. All patients provided written, informed consent prior to participating in this study. The Lawson Health Research Institute Research Ethics Board at Western University in London, Ontario, Canada approved the study.

The auto-injector needle length was considered adequate if STMD_max_ was ≤15.2 mm. Our primary goal was to determine the proportion of patients with a STMD_max_ >15.2 mm since these subjects are at risk of receiving epinephrine subcutaneously, rather than intramuscularly as currently recommended. Hence, the primary outcome variable was the proportion of subjects with a STMD_max_ >15.2 mm. A secondary variable was the proportion of women with a STMD_max_ >15.2 mm. The participants were divided into two groups based on whether their STMD_max_ was ≤15.2 mm or >15.2 mm. Baseline characteristics of participants were analyzed using the Student’s t-test/Mann Whitney U test for continuous variables and Chi-square/Fisher’s exact test for categorical variables. Multivariable linear regression analysis was used to identify factors, including sex, age, weight, height, BMI, that were significantly associated with a STMD_max_ >15.2 mm in both the entire patient cohort and in females. The percent compression of the STMD and the proportions of female patients with a STMD_max_ less than 15.2 mm, 20 mm, 25 mm and 30 mm were also assessed.

## Results

**Figure 2:**
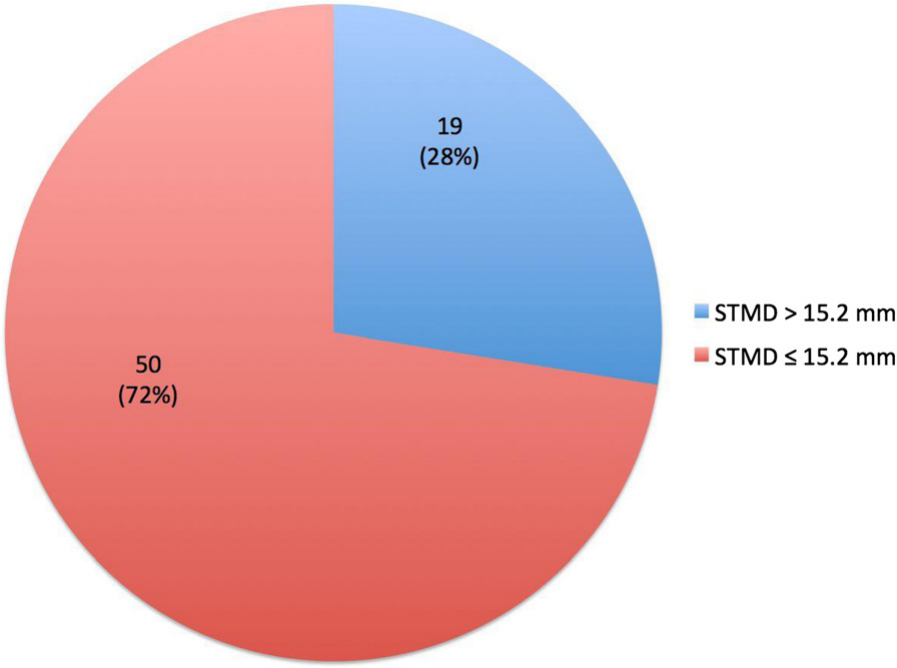
**Proportion of women with STMD**_**max **_**less than/equal to or >15.2 mm.**

The STMD_max_ was used to determine if needle length was adequate, as this most closely mimics patient characteristics at the time of administration of the epinephrine auto-injector. The subjects ranged from 18 to 72 years of age. Nineteen of the 100 patients included in this study (19%) had a STMD_max_ >15.2 mm; 28% of women had a STMD_max_ >15.2 mm (Figure 2). None of the men in this study had a STMD_max_ >15.2 mm.

**Figure 3:**
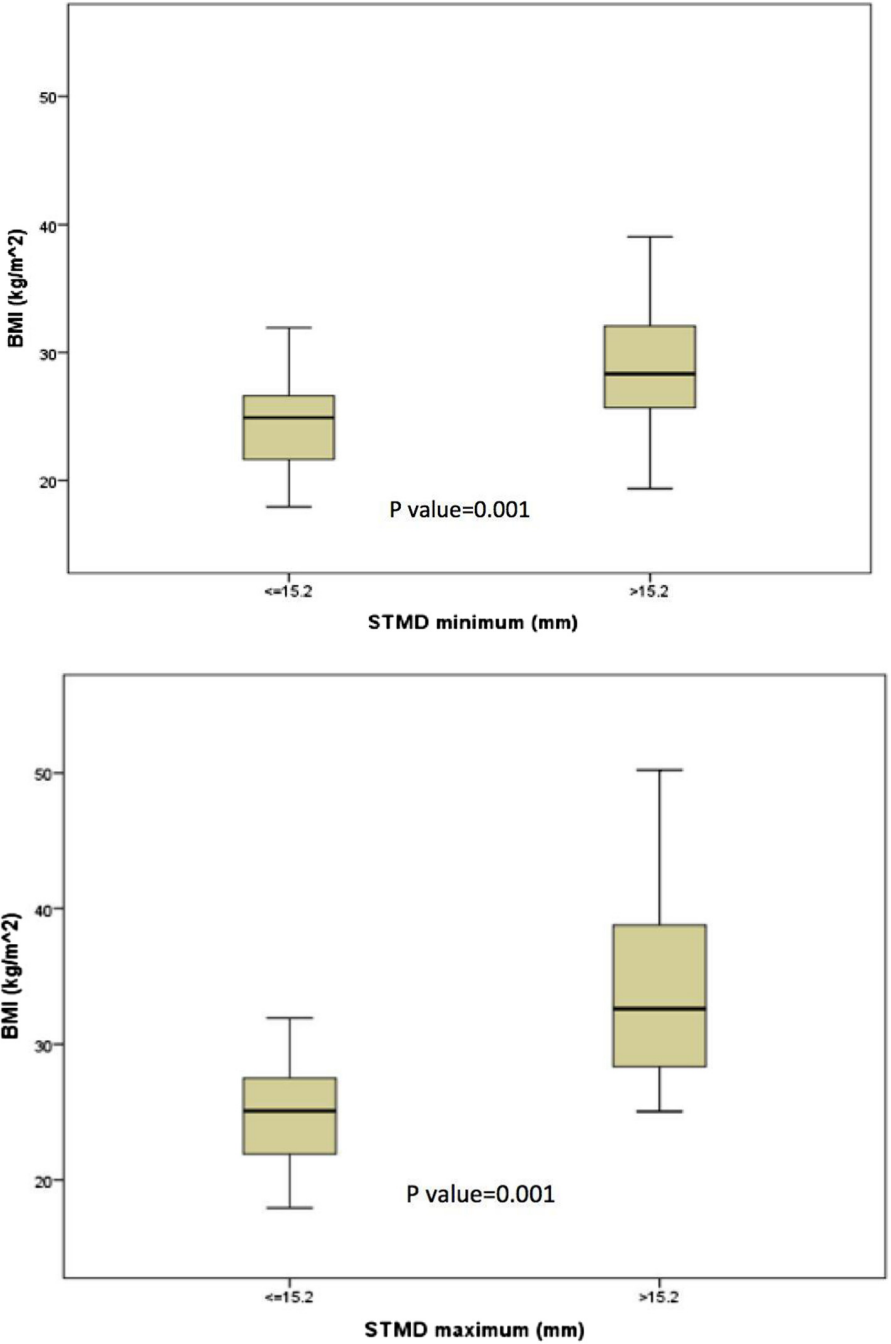
**Box plots showing the differences in BMI between the two comparison groups.** BMI: body mass index; STMD: skin-to-muscle depth.

Sex, weight, height, BMI, and mean STMD differed significantly between subjects with a STMD_max_ less than/equal to vs. >15.2 mm (all p < 0.05, Table 1). Weight and BMI were higher, and height was lower, in those subjects with a STMD_max_ >15.2 mm. In women, weight, BMI and mean STMD differed significantly between the two comparison groups (all p < 0.05, Table 2). Multivariable linear regression analysis found BMI (p = 0.001) to be significantly associated with STMD at both minimal and maximal pressure after adjusting for age (Figure 3). Mean change in STMD with pressure was 11% for all subjects and 16% for women. This change was less than the 25% estimated in a previous study [8]. The percentage of women with a STMD_max_ ≤15.2 mm, <20 mm, <25 mm and <30 mm was 72%, 91%, 96% and 99%, respectively. Only one woman had a STMD_max_ ≥30 mm (Table 3).

**Table 1:** Baseline characteristics of study population

**Characteristic**	**Total, n = 100**	**Patients with STMD minimum**		P value*	**Patients with STMD maximum**		P value*
		≤15.2 mm, n = 68	>15.2 mm, n = 32		≤15.2 mm, n = 81	>15.2 mm, n = 19	
Age (years), mean (SD)	37 (14)	35 (15)	41 (13)	0.01	36 (14)	42 (14)	0.06
Males, n (%)	31 (31)	31 (45)	0 (0)	0.00001	31 (37)	0 (0)	0.001
Caucasian, n (%)	89 (89)	59 (87)	30 (93)	0.495	71 (88)	18 (95)	0.685
Weight (kg), mean (SD)	76 (19)	73 (19)	83 (18)	0.014	74 (18)	89 (19)	0.003
Height (m), mean (SD)	1.7 (0.1)	1.69 (0.1)	1.64 (0.1)	0.020	1.69 (0.1)	1.64 (0.1)	0.02
BMI (kg/m^2^), mean (SD)	27 (6)	25 (5)	31 (7)	0.001	26 (5)	33 (8)	0.0001
STMD max (mm), mean (SD)	11 (6)	8 (3)	17 (4)	0.0001	9 (3)	19 (4)	0.0001
STMD min (mm), mean (SD)	13 (7)	9 (3)	21 (6)	0.0001	11 (5)	23 (7)	0.0001
STMD% compression, mean (SD)	11 (10)	11 (10)	18 (10)	0.002	13 (11)	15 (9)	0.41

*P-value calculated by Student’s t test or Chi square test/Fisher’s exact test.

SD: standard deviation; BMI: body mass index; STMD_min_: skin-to-muscle depth with minimal pressure; STMD_max_: skin-to-muscle depth with maximal pressure.

**Table 2:** Baseline characteristics of females in the study

**Characteristic**	**Total, n = 69**	**Patients with STMD minimum**		P value*	**Patients with STMD maximum**		P value*
		≤15.2 mm, n = 37	>15.2 mm, n = 32		≤15.2 mm, n = 50	>15.2 mm, n = 19	
Age (years), mean (SD)	37 (13)	33 (12)	41 (13)	0.009	35 (12)	43 (14)	0.03
Caucasian, n (%)	61 (88)	31 (82)	30 (94)	0.27	43 (86)	18 (95)	0.29
Weight (kg), mean (SD)	73 (17)	64 (12)	83 (18)	0.001	67 (12)	89 (19)	0.001
Height (m), mean (SD)	1.6 (0.1)	1.62 (0.1)	1.64 (0.1)	0.16	1.63 (0.1)	1.64 (0.1)	0.71
BMI (kg/m^2^), mean (SD)	27 (7)	24 (4)	31 (7)	0.0001	25 (4)	33 (8)	0.001
STMD max (mm), mean (SD)	13 (5)	10 (2)	17 (4)	0.0001	11 (3)	20 (4)	0.0001
STMD min (mm), mean (SD)	16 (7)	11 (2)	21 (6)	0.0001	13 (4)	23 (7)	0.0001
STMD% compression, mean (SD)	16 (11)	14 (11)	18 (10)	0.11	16 (11)	15 (9)	0.53

*P-value calculated by Student’s t test or Chi square test/Fisher’s exact test.

SD: standard deviation; BMI: body mass index; STMD_min_: skin-to-muscle depth with minimal pressure; STMD_max_: skin-to-muscle depth with maximal pressure.

**Table 3:** **Proportion of females with STMD**_**max **_**at different thresholds**

**Groups**	**Number of women, n (%)**
≤15.2 mm	50 (72%)
<20 mm	63 (91%)
<25 mm	66 (96%)
<30 mm	68 (99%)
≥30 mm	1 (1%)

## Discussion

The results of this study demonstrate that although the needle length of a standard epinephrine auto-injector is likely adequate for intramuscular delivery of epinephrine in many patients at risk of anaphylaxis, it does not appear to be adequate for a significant proportion of women. These findings are consistent with those of a 2005 study conducted in patients who had undergone CT of their thighs for other medical reasons, as well as a 2013 study of low-acuity patients in the emergency department setting [8,9]. The former study assessed the CT scans of the legs of patients who had been investigated for deep vein thrombosis with or without pulmonary embolus [9]. A number of CT scans (180) were retrospectively reviewed and the first 100 were included in the study if the patient’s height and weight were also available. No images or measurements of the STMD with compression were completed in these subjects. Based on testing of one male and one female subject, the investigators estimated that there would be a 25% reduction in the STMD with 8 pounds of pressure applied to the leg. Also, the investigators used a needle length of 14.3 mm to determine adequacy of the epinephrine auto-injector for intramuscular administration. The 2013 study conducted in the emergency department setting was also performed in patients who were not at risk of anaphylaxis [8]. Subjects were recruited prospectively, in a non-consecutive fashion, only during the time when study investigators were available. Ultrasound measurements were completed with and without pressure to simulate the pressure required to trigger the auto-injector device. In that study a needle length of 15.9 mm was used. Also, the ultrasound images were taken at the spot where the hand laid to rest on the lateral thigh, following the directions in the EpiPen^®^ product monograph [6]. It is important to note that our study is the first to be completed in adult patients with food allergy who were at risk of anaphylaxis. All patients required an epinephrine auto-injector and were recruited into the study consecutively. This is also the first study to assess the impact of applying pressure on the site of auto-injector application in order to measure STMD_max_ at the mid-anterolateral thigh in these at-risk patients. We believe this is the best method to assess whether needle length of the auto-injector is adequate for intramuscular administration at the proper site of injection [1,3]. We used the needle length that is published in the EpiPen^®^ patent at 15.2 mm [7]. We identified that, with pressure, the subcutaneous space was compressed by only 11%, which is significantly less than the estimated 25% in the study by Song et al. [8].

Two studies, one completed in adults and one in children, have suggested that intramuscular administration leads to higher serum levels of epinephrine more rapidly than subcutaneous injections [4,5]. In fact, intramuscular administration of epinephrine into the thigh has become the standard recommended treatment for anaphylaxis due to the results of the adult study [5], which included 13 men in a 6-way crossover design. However, it is important to note that ultrasound was not used to confirm whether epinephrine was truly delivered to the appropriate tissue compartment, and no female subjects were included in this study. It is possible that the pharmacokinetics and pharmacodynamics of epinephrine may differ between men and women. Future studies should include women to determine if such gender differences are present. Also, there are no human randomized controlled studies assessing the effects of epinephrine in the clinical setting of anaphylaxis. These studies would be practically challenging and may be considered unethical taking into account the studies noted above [4,5].

Similar to the findings of Bhalla et al. [8] and Song et al. [9] discussed earlier, our study found that only women were at risk of subcutaneous injections of epinephrine. Certainly, however there will be both men and women with anaphylaxis who will have a STMD_max_ greater than the needle lengths of the currently available epinephrine auto-injectors. Importantly, our study is the first to use the EpiPen^®^ needle length that is published in the formal patent submission. Ideally, ultrasounds should be performed in all patients prescribed auto-injectors to assist in identifying patients at risk of subcutaneous injections. For these patients, an alternative method of epinephrine injection could be to provide vials of epinephrine and syringes with longer needles. However, this approach may potentially lead to incorrect dosing and slower delivery of the epinephrine injection [11]. Modifying the method of injecting the auto-injector into the thigh may lead to deeper injections. For example, increased compression or more “hand swing” may trigger the auto-injector to deliver epinephrine more deeply [10]. The availability of auto-injector devices with longer needle lengths would be another potential solution to this problem. In the United Kingdom, there is an epinephrine device available which has a 25-mm long needle [12]. At this needle length, epinephrine would be injected intramuscularly in 96% of our female subjects. If the auto-injector had a 30-mm needle length, 99% of women would appropriately receive the epinephrine intramuscularly (Table 3). However, it is likely that at a certain length, the needle would be too long for some patients, leading to injection of the epinephrine into the bone.

The clinical implications of failure to deliver epinephrine intramuscularly are unknown. Our major concern is that patients with a STMD_max_ greater than the auto-injector needle length will not attain adequate epinephrine levels rapidly enough to treat anaphylaxis, thereby increasing the risk of death. At present, there are no published studies or case reports to confirm this concern. Nonetheless, studies assessing deaths from anaphylaxis have identified that many deaths occur in women, and that some of these women were at risk of having a STMD_max_ greater than 15.2 mm [2]. Also, a media report from 2011 suggests that a longer epinephrine auto-injector needle length may have saved the life of a 19-year-old female in England who died of anaphylaxis from food allergy [13]. The European Medicines Agency is currently reviewing epinephrine auto-injectors to assess whether the needle length is appropriate for intramuscular delivery [14].

The main strengths of this study were that the patients included adults at risk of anaphylaxis, the subjects were recruited consecutively, the STMD_max_ was used as the primary variable, the proper length of the EpiPen^®^ needle was used based on the patent submission for the product and ultrasound measurements were taken in the recommended location for epinephrine auto-injector application. The findings of this paper confirm that a significant number of women at risk of anaphylaxis are at risk of injecting epinephrine into the subcutaneous space and, therefore, may not be receiving the proper benefits of this potentially life-saving medication.

Our study has some limitations. Firstly, we only estimated the pressure and the depth of using an epinephrine auto-injector with the ultrasound probe. A more accurate method of applying pressure would be to actually measure the pressure required to trigger an auto-injector and then to perform the ultrasound with a similar pressure. However, even with consistent pressure applied, the surface areas of the ultrasound probe and the epinephrine auto-injectors are different. This may lead to varying depths of compression. Also, our estimate of compression will likely be different than the “real-life” compression that occurs when a patient actually uses an auto-injector. Patients will most likely use variable levels of force when self-administering the auto-injector. As well, the ultrasound measurements were completed with subjects in the standing position only as this is the recommended position in the images of the EpiPen^®^ product monograph. However, the EpiPen^®^ could be injected in the lying or sitting positions in differing clinical situations. Future studies could complete ultrasound measurements in these different body positions.

Secondly, one unblinded physician performed all of the ultrasounds in this study. Although this may lead to more consistent test results, it may have also lead to observer bias among the tests that were completed. Thirdly, the study was performed in one clinic in Southern Ontario, Canada. Although the patients in this study are likely representative of adults with food allergy in Southern Ontario, they may not be representative of patients from other geographic and socioeconomic areas. This may explain the difference between our findings and those of Bhalla et al. [8] who found that 31% of their entire patient cohort and 54% of their female subjects were “failure risks” (i.e., patient muscle depth exceeded the 15.9-mm length of the auto-injector needle). It is important to note that this latter study was conducted in Ohio, USA, where the prevalence of obesity is approximately 30% [15]. Ideally, a multicentre study including patients from various geographic and socioeconomic areas would be the preferred approach for studying this issue.

Finally, we did not formally assess whether another available epinephrine auto-injector, Allerject^®^, with a needle length of 15.7 mm would have comparable results. We did identify two patients in our study that would have had the Allerject^®^, but not the EpiPen^®^, auto-injector reach the intramuscular space. As future products become available, the needle lengths, real injection depths and true function of these devices should be considered before they are prescribed.

## Conclusions

To our knowledge, this is the first study evaluating the adequacy of the epinephrine auto-injector needle length to deliver epinephrine intramuscularly in adult patients at risk of anaphylaxis. Our study suggests that in up to 19% of adult patients, and in approximately 28% of adult females, the commercially available epinephrine auto-injectors will potentially not reach the intramuscular space. In these patients, the epinephrine auto-injectors may not be ideal for the rapid treatment of anaphylaxis. Further research to determine the clinical implications of inadequate needle lengths of epinephrine auto-injectors is recommended.

## Competing interests

HK has been on advisory boards for Sanofi Canada and the speakers’ bureau for Pfizer Canada. GT, LK, IN, RP, JC, AD declare that they have no competing interests.

## Authors’ contributions

GT, LK, RP, JC, and HK were responsible for the conception and design of the study. LK and RP were responsible for the acquisition of the data. IN and AD were responsible for data analysis. GT, LK and IN drafted the manuscript. All of the authors contributed substantially to the interpretation of the data, critically revised the manuscript for important intellectual content, approved the final version submitted for publication and agree to act as guarantors of the work.
